# Cost-Effectiveness Analysis of Hepatitis E Vaccination Strategies for Swine Workers

**DOI:** 10.1155/tbed/9371055

**Published:** 2025-03-10

**Authors:** Fengge Wang, Lu Zhou, Yihan Lu

**Affiliations:** ^1^Shanghai Institute of Infectious Disease and Biosecurity, Fudan University, Shanghai, China; ^2^Department of Epidemiology, Ministry of Education Key Laboratory of Public Health Safety, School of Public Health, Fudan University, Shanghai, China

**Keywords:** cost-effectiveness analysis, hepatitis E vaccine, swine workers, vaccination

## Abstract

Hepatitis E virus (HEV) is endemic in China, with swine as the most common reservoir. It poses a zoonotic public health risk to swine workers. This study evaluated the cost-effectiveness of hepatitis E vaccination for this high-risk group in China. A decision tree-Markov model was utilized to evaluate the cost-effectiveness of two hepatitis E vaccination strategies, without or following screening, for swine workers aged 16–60 in China from societal perspectives, compared to no vaccination. We calculated HEV-related cases and deaths averted, quality-adjusted life years (QALYs) gained, and incremental cost-effectiveness ratios (ICERs) with a willingness-to-pay (WTP) threshold of GDP per capita. A sensitivity analysis was conducted. Additionally, we stimulated the scenarios of fully receiving 3-dose schedule, partially receiving 3-dose schedule, and fully receiving 2-dose schedule. Both hepatitis E vaccination strategies significantly reduced HEV-related cases and deaths compared to no vaccination. ICERs were estimated to be USD 11,428.16 and 9830.71/QALY averted for vaccination without and following screening, respectively, both lower than GDP per capita (USD 12,325.24, 2023). Furthermore, one-way sensitivity analysis identified the discount rate, utility in asymptomatic cases, and probability of symptomatic infection as crucial factors affecting ICER. Probabilistic sensitivity analysis (PSA) showed a 47.5% cost-effectiveness probability for hepatitis E vaccination following screening, compared to 52.5% for no vaccination. Notably, vaccination following screening was cost-ineffective after age 40 and at a price of USD 138.0/dose. Additionally, fully receiving 2-dose and partially 3-dose schedules were cost-effective, regardless of hepatitis E vaccination without or following screening strategies, while fully receiving 3-dose schedule was cost-ineffective with the vaccination without screening strategy. Hepatitis E vaccination following screening would be optimal for swine workers in China. Vaccination starting at an earlier age and lower vaccine prices can improve the cost-effectiveness. Additionally, 2-dose schedule may be recommended during a hepatitis E outbreak to achieve cost-effectiveness.

## 1. Introduction

Hepatitis E virus (HEV) is the causative agent of hepatitis E, which is currently recognized as a significant global public health concern, particularly in low- and middle-income countries [1]. According to the World Health Organization (WHO), ~20 million people are infected with HEV globally each year, resulting in 3.3 million symptomatic cases and 70,000 deaths related to HEV infection [2]. Among the four common genotypes of HEV (HEV-1 to HEV-4), HEV-1 and HEV-2 exclusively infect humans and are primarily transmitted via fecal–oral route, typically occurring in areas with poor sanitation [3]. HEV-3 and HEV-4 are zoonotic genotypes that infect both humans and a wide range of animal hosts. Swine is the most common host globally and in China. HEV prevalence in humans is notably high, with a meta-analysis showing that about 12.5% of the global population has been infected [4]. Moreover, clinical symptoms of HEV infection range from asymptomatic cases to severe hepatitis. Most infected individuals, especially the young and immunocompetent, show no significant symptoms or have self-limiting hepatitis with a low mortality rate. However, in the elderly, pregnant women, and those with chronic liver disease, HEV infection can cause acute or chronic hepatitis and various extrahepatic complications, including neurological syndromes, leading to severe outcomes [5].

Swine and pork products play a critical role in HEV transmission [6, 7]. Serological testing in swine detected anti-HEV IgG antibodies in 57.53% of samples, with positive cases found in 96% of the farms [8]. Given the scale of China's pork industry—slaughtering 699.95 million swine and producing 55.41 million tons of pork in 2022—various swine-related occupations, from breeding to sales, are at risk of HEV exposure [9]. A meta-analysis found that in China, the seroprevalence of anti-HEV IgG among those swine workers was 48.41% (95% CI: 40.02–56.85) [10]. Similarly, a study conducted in Guangzhou, China, found that HEV IgG seroprevalence among swine workers was 47.0%, compared to 26.1% in the general population [11]. Moreover, routine swine trade has facilitated the spread of HEV across different regions in China, complicating its epidemic characteristics. Therefore, implementing strict protective measures for swine workers is essential to reduce occupational HEV transmission.

Hepatitis E vaccination has proven to be an effective and safe method for preventing HEV infection. In 2012, the hepatitis E vaccine (Hecolin) was licensed and introduced in China. The vaccine has effective and stable neutralizing epitopes that can induce a more efficient humoral immune response, which may provide long-term protection against hepatitis E infection [12–14]. Current health economic evaluations of hepatitis E vaccination strategies for the elderly, women of childbearing age, and chronic hepatitis B patients indicated that hepatitis E vaccination combined with screening strategy was the most cost-effective strategy, compared to universal vaccination or no vaccination [15–18].

However, it remains limited in the understanding of hepatitis E vaccination among swine workers. This study aimed to conduct a health economic evaluation of hepatitis E vaccination strategies in this high-risk group. A decision tree–Markov model was used to assess the cost-effectiveness of two vaccination strategies: vaccination without screening and vaccination following screening, and no vaccination.

## 2. Methods

### 2.1. Model Design

We constructed a decision tree–Markov model to simulate the costs and effects of hepatitis E vaccination without screening, vaccination following screening, and no vaccination strategies using a hypothetical cohort of 100,000 individuals. The swine workers in this study referred to persons who work with swine, including swine farmers, butchers, meat processors, pork retailers, and veterinarians [19]. In the simulation, the entire cohort was initially categorized into different decision tree branches based on their immune status against HEV infection and intent to receive hepatitis E vaccination and subsequently entered different Markov states.

**Figure 1:**
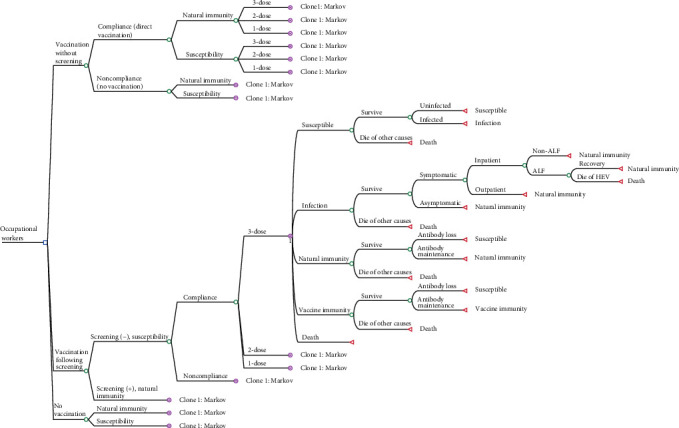
Decision tree-Markov model of hepatitis E vaccination for swine workers.

The overview of the model structure is presented in Figure 1. We developed the hepatitis E vaccination strategies as follows: (1) Vaccination without screening: individuals in the cohort received the hepatitis E vaccine based on their vaccination intent. Since vaccine efficacy did not achieve 100%, those who did not successfully acquire immunity entered the Markov model in either natural immunity or susceptibility states. (2) Vaccination following screening: only individuals who tested negative for anti-HEV antibodies received the vaccine based on vaccination intent. (3) No vaccination: individuals in the cohort did not receive the vaccine and followed the natural history of HEV infection, serving as the control group. Individuals willing to receive the hepatitis E vaccine may receive a 1-dose, 2-dose, or 3-dose schedule. According to the “Consensus on prevention and treatment of hepatitis E,” the recommended hepatitis E vaccination schedule was three doses at 0, 1, and 6 months [20].

Based on the natural course of hepatitis E, we designed five distinct Markov states: susceptibility, infection, natural immunity, vaccination immunity, and death. During the incubation period, infected individuals are asymptomatic or experience mild symptoms. Then, depending on disease severity, they may seek outpatient care or require hospitalization. The illness usually resolves within a few weeks without chronic progression in immunocompetent individuals. However, in rare cases, it may deteriorate into acute liver failure (ALF), requiring intensive medical intervention. Following recovery, individuals develop natural immunity, which provides protection against reinfection for an uncertain duration. Within the Markov model, they could be infected with HEV each year (representing a Markov cycle). The probability of each Markov state depended on the transition probabilities. The cycles ended at the retirement age of 60.

### 2.2. Parameters Input

In the model, base case values, ranges, and distributions of the parameters were estimated through a literature review and expert consultation (Table 1).

#### 2.2.1. Probability Parameters

Probability parameters included HEV infection rate, symptomatic infection, hospitalization, progression to ALF, mortality rate, natural immunity rate, waning rate of natural immunity, vaccine efficacy, and intent to receive hepatitis E vaccine. HEV infection rates among livestock workers in various regions were used as reference values for the susceptibility-to-infection transition rates in this population [21]. Based on WHO surveys, an estimated 20 million people globally were infected with HEV annually, and ~3.3 million were symptomatic cases. Thus, these data were used to estimate the symptomatic rate of HEV infection [1, 2, 22]. Parameters for disease progression following HEV infection, including hospitalization and ALF, were sourced from recent epidemiological studies and reviews [23, 24]. Age-specific mortality rates were derived from the 2020 China National Census data [25]. Natural immunity rates for swine farmers and slaughterhouse workers of different age groups were derived from recent epidemiological studies and meta-analyses on IgG antibodies conducted in China [10, 26, 27]. Furthermore, the waning of natural immunity in the population was derived from long-term follow-up studies and monitoring of antibody levels in asymptomatic individuals [28]. Vaccine efficacy, decay of vaccine-induced immunity, and coverage of different vaccine dosing schedules were obtained from long-term clinical studies of the hepatitis E vaccine and previous studies on hepatitis A in China and Bangladesh [12–14, 29, 30]. A survey in Yantai City, China, found that 54.0% of middle-aged and elderly participants were willing to receive the hepatitis E vaccine [32]. Furthermore, the vaccination intent would be as high as 80.0% when participants knew their screening results [29, 31]. Additionally, due to the high quality of current detection methods, this study did not consider false positives and negatives from screening methods. All rates were then converted to probabilities using the formula embedded in the software: *P = 1−e^−^*^*rt*^.

#### 2.2.2. Cost Parameters

The costs included both vaccine-related and disease-related expenses. Vaccine-related costs comprised serologic testing for HEV immunity, vaccine price, vaccine administration, and indirect costs associated with vaccination, such as transportation and lost wages [34, 35]. The vaccine price was determined based on government procurement prices [33]. Disease-related expenses included outpatient and inpatient visits for diagnosis and treatment of HEV infection and costs associated with HEV-related complications and mortality. These expenses were derived from recent field surveys conducted among hepatitis E patients in Jiangsu and Guangdong Provinces, China [36–38]. We excluded the costs of vaccination-related adverse events and sequelae because the hepatitis E vaccine Hecolin was well-tolerated, with few mild side effects that did not require medication [13, 14]. Additionally, the GDP per capita used in the analysis was CNY 89,358 in 2023, according to the National Bureau of Statistics of China [39]. All costs were then converted to USD in March 2024 using the exchange rate of USD1 = CNY7.2465 [44]. The willingness-to-pay (WTP) threshold was set at the GDP per capita, USD12,325.24, following standard recommendations for economic evaluations.

#### 2.2.3. Utility Parameters

Utilities were attached to each of the Markov states. Quality-adjusted life year (QALY) was determined by multiplying the number of life years by the utility value that represented the quality of life during these years. Due to the absence of life-quality studies for asymptomatic HEV infection, we estimated the utility based on the findings in the 1990–2010 hepatitis E surveys in China and other field studies [40, 41]. The utilities of outpatient and inpatient states were taken from field studies in Jiangsu Province [36, 38]. The utility of ALF was estimated based on hepatitis B disease states [42]. A 5% discount rate was applied to both effects and costs.

### 2.3. Statistical Analysis

#### 2.3.1. Base Case Analysis

We conducted a cost-effectiveness analysis of two hepatitis E vaccination strategies, compared to no vaccination, by using the decision tree–Markov model. In this model, health outcomes were defined as a cumulative number of hepatitis E outpatient and inpatient cases, ALF, deaths, and QALYs over the entire time horizon for each vaccination strategy. Economic outcomes included the costs avoided under those vaccination strategies. The incremental cost-effectiveness ratio (ICER) was calculated to determine the cost required to gain one unit of health benefit. An ICER of less than GDP per capita indicated a cost-effective strategy.

#### 2.3.2. Sensitivity Analysis

We conducted one-way and probabilistic sensitivity analyses (PSAs) to evaluate the uncertainty of parameters in the model. Key factors were analyzed using one-way sensitivity analysis, with results presented in tornado diagrams. PSA was conducted through Monte Carlo simulations, with 1000 random samplings under various probability distributions. Moreover, the cost-effectiveness acceptability curve showed the probability that different vaccination strategies were cost-effective, compared to no vaccination, at various WTP thresholds. The incremental cost-effectiveness scatterplots represented the relationship between incremental cost and incremental effectiveness of different vaccination strategies. The decision tree–Markov model was developed using TreeAge Pro 2022 software (TreeAge Software, Inc., MA, USA).

#### 2.3.3. Scenario Analysis

The HEV vaccine is currently licensed for individuals aged 16 and above. According to the Labor Law of the People's Republic of China, the legal age for occupational groups ranges from 16 to 60 years [43]. To assess the health and economic effects of swine workers' hepatitis E vaccination starting at different ages, we classified the cohort into five age groups (16–60, 20−60, 30–60, 40−60, and 50–60 years) and compared the cost-effectiveness by age.

Scenario analysis of vaccine prices enables a comprehensive evaluation of various impacts due to price change, supporting budget planning and resource allocation optimization. Therefore, we set up scenarios with multiple HEV vaccine prices and conducted a comparative analysis to explore their costs and effectiveness.

The hepatitis E vaccination schedule consists of three doses, administered at 0, 1, and 6-month intervals. However, the HEV vaccine is relatively expensive, and studies have shown that two doses may also provide good protection [13]. Recently, a hepatitis E outbreak occurred in eastern Chad from January to April, with 2092 suspected cases and seven deaths notified [45]. The WHO [46] Strategic Advisory Group of Experts (SAGE) on Immunization recommended the use of an off-label 2-dose schedule (at 0 and 1-month intervals) instead of the 3-dose schedule during outbreaks in fragile, conflict-affected, and vulnerable (FCV) settings. Therefore, this study performed a scenario analysis of different vaccination schedules.

## 3. Results

### 3.1. Vaccination Cost

This study showed that with hepatitis E vaccination without a screening strategy, ~151,000 doses were administered to 100,000 swine workers in China in 2024, resulting in a total cost of USD 18.11 million. With the vaccination following the screening strategy, 180,500 doses were administered, costing USD 22.06 million, with an incremental cost of USD 3.95 million (Table 2).

### 3.2. Cost-Effectiveness Analysis

Of the two hepatitis E vaccination strategies and no vaccination, the number of outpatient cases with HEV infection was estimated to be the highest, which was 6802, 6111, and 9932, respectively (Table 3). Compared to no vaccination, hepatitis E vaccination without screening strategy reduced the number of outpatient cases by 31.5%, and vaccination following screening strategy reduced them by 38.5%.

Moreover, the base case analysis revealed that the total cost of hepatitis E vaccination without screening strategy was USD 22,370,398.60, with an ICER of USD 11,428.16/QALY, less than the GDP per capita (USD 12,325.24, 2023). In contrast, the vaccination following the screening strategy had a lower ICER of USD 9830.71/QALY, though it had a higher cost of USD 25,383,253.82, indicating better cost-effectiveness. Screening identifies infected or naturally immune individuals, avoiding unnecessary vaccination while also increasing vaccination willingness and coverage, thereby optimizing vaccine allocation, reducing healthcare costs, and improving cost-effectiveness. Therefore, the vaccination following the screening strategy was recommended to maximize health protection and cost-effectiveness despite its higher cost.

### 3.3. Sensitivity Analysis

#### 3.3.1. One-Way Sensitivity Analysis

**Figure 2:**
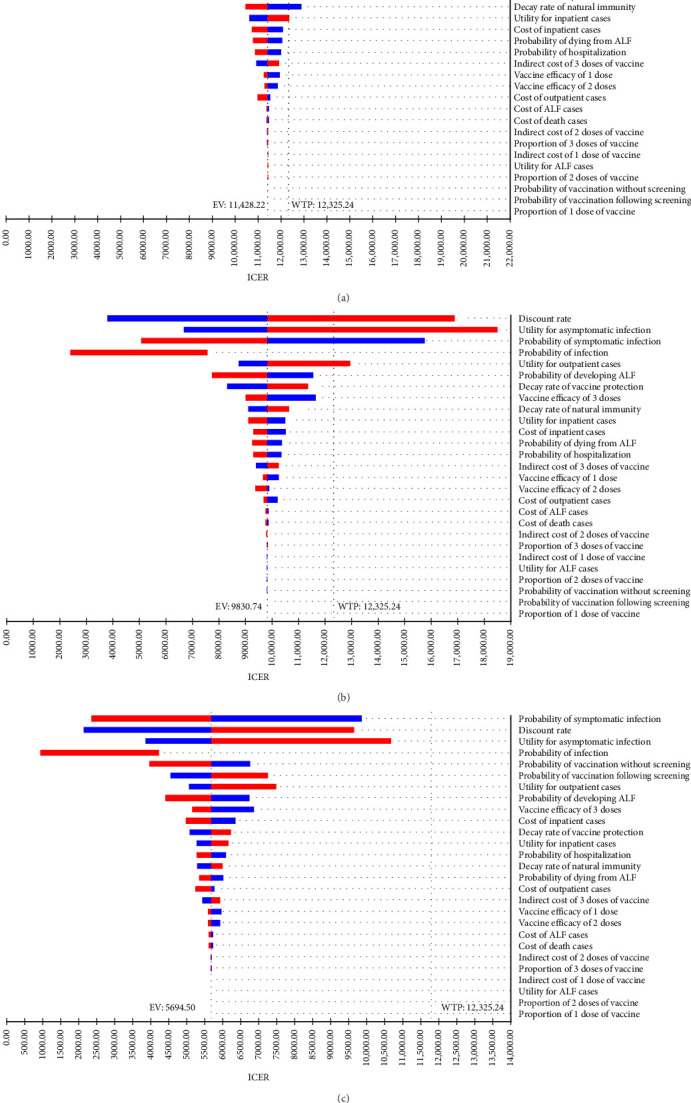
Tornado diagram for one-way sensitivity analysis of hepatitis E vaccination cost-effectiveness in swine workers. (A) Vaccination without screening vs. no vaccination, (B) vaccination following screening vs. no vaccination, and (C) vaccination without screening vs. vaccination following screening. The horizontal axis represents the range of ICER changes. Red bars to the left of the baseline and blue bars to the right indicate that as the factor decreases, the ICER increases. Conversely, blue bars to the left of the baseline and red bars to the right indicate that as the factor increases, the ICER increases. The length of each bar indicates the extent to which each factor influences the ICER. ICER, incremental cost-effectiveness ratio.

The one-way sensitivity analysis indicated the impact of each parameter on the ICER estimation. The ICER was most influenced by multiple factors, including the discount rate, utility of asymptomatic cases, probability of symptomatic infection, and utility of outpatient cases, with ICER ranging from nearly USD 3000/QALY to over USD 20,000/QALY, depending on the range of each parameter when comparing hepatitis E vaccination without screening strategy to no vaccination (Figure 2A). It was similar for the vaccination following the screening strategy (Figure 2B). In general, a higher discount rate, higher utility of asymptomatic cases, and higher utility of outpatient cases led to an increased ICER, while a higher probability of symptomatic infection resulted in a reduced ICER. The other parameters had minimal impact on the effects of hepatitis E vaccination strategies.

#### 3.3.2. PSA

**Figure 3:**
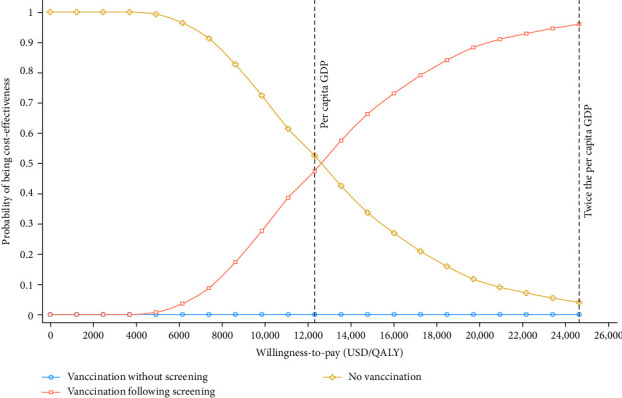
Cost-effectiveness acceptability curves of two hepatitis E vaccination strategies and no vaccination in swine workers.

By using PSA, we further assessed the possible outcomes of parameters within their ranges of variation in the base case scenario. When the WTP threshold was set at GDP per capita (USD 12,325.24, 2023), hepatitis E vaccination following screening strategy had a cost-effectiveness probability of around 47.5%. At a WTP threshold of two times GDP per capita, this probability was 95.3% (Figure 3). It demonstrated a positive association between the WTP threshold and the cost-effectiveness of hepatitis E vaccination following screening strategy. In contrast, the vaccination without screening strategy had an acceptability probability close to 0 at all WTP thresholds. This suggested that no vaccination strategy was optimal at lower WTP levels, while the vaccination following screening strategy showed higher cost-effectiveness at higher WTP levels.

**Figure 4:**
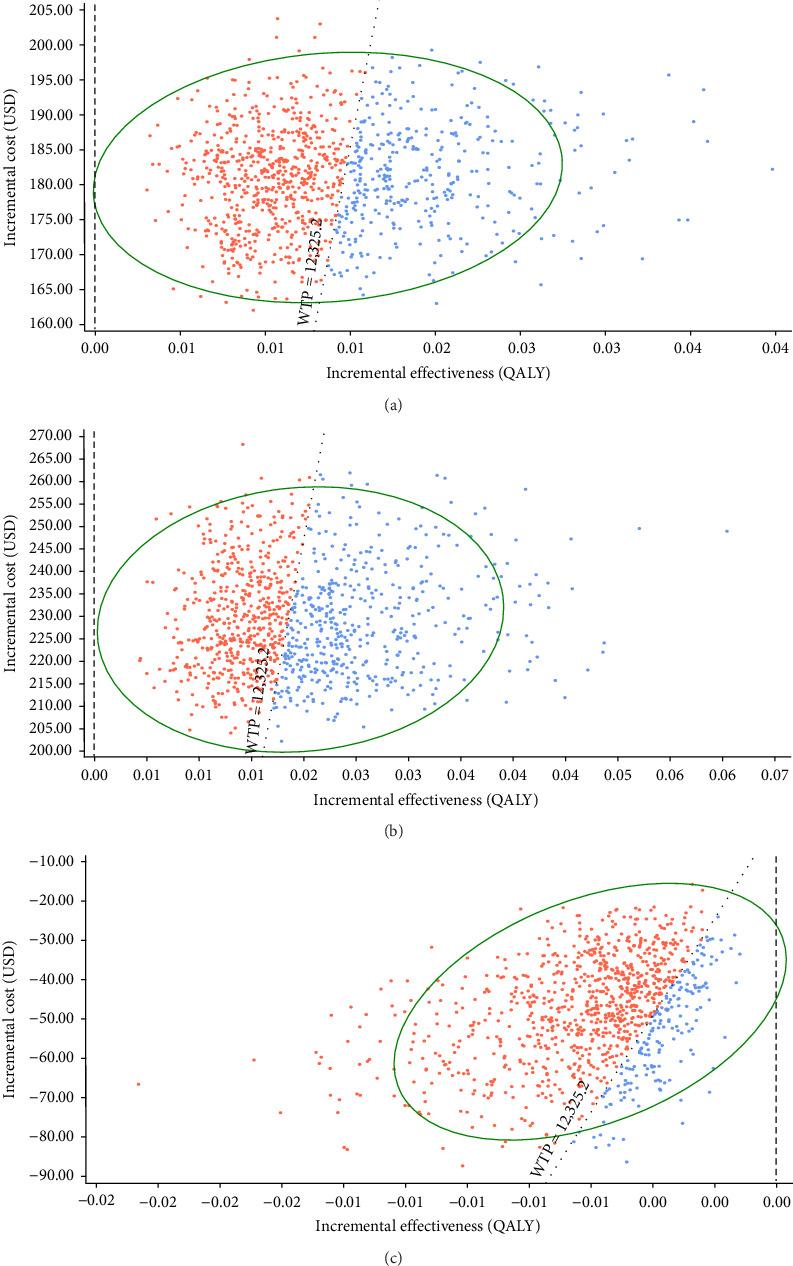
Incremental cost-effectiveness scatterplots of the cost-effectiveness probability sensitivity analysis for hepatitis E vaccination strategies. (A) ICER for vaccination without screening vs. no vaccination, with 37.1% of points below the WTP line. (B) ICER for vaccination following screening vs. no vaccination, with 47.8% of points below the WTP line. (C) ICER for vaccination without screening vs. vaccination following screening, with 84.8% of points above the WTP line. ICER, incremental cost-effectiveness ratio; WTP, willingness-to-pay.

The incremental cost-effectiveness scatterplots showed the incremental effect remained stable while the incremental cost was more uncertain. In most cases, hepatitis E vaccination resulted in higher incremental costs compared to no vaccination (Figure 4A,B). The proportion of scatter points above the WTP threshold was comparable to the proportion below it. This indicated that, compared to no vaccination, the cost-effectiveness advantages of the vaccination strategies were not pronounced due to the wide range of parameter variability. However, most of the scatter points were above the WTP threshold line, indicating that the vaccination following screening strategy was advantageous in terms of benefits compared to the vaccination without screening strategy (Figure 4C).

### 3.4. Scenario Analyses

#### 3.4.1. Vaccination Age

This study showed an association between the time span and number of avoided HEV-related cases (Figure S1). Hepatitis E vaccination starting at an earlier age prevented the disease occurrence more effectively and cost-effectively. For the age group of 16–60 years, the vaccination without screening and vaccination following screening strategies prevented ~31.5% and 38.5% of HEV-related cases, respectively (Table S1). The ICERs of both hepatitis E vaccination strategies were below GDP per capita. Furthermore, as the vaccination starting age increased, the number of cases avoided decreased while the ICER gradually increased. When the vaccination started after age ≥30, the ICER for the vaccination without screening strategy exceeded the GDP per capita. Similarly, when the vaccination started after age ≥40, the vaccination following the screening strategy was no longer cost-effective (Table S2).

#### 3.4.2. Vaccine Price

Similar patterns were observed with multiple vaccine prices. At a lower vaccine price of USD 69.00, the ICER for hepatitis E vaccination without screening and vaccination following screening strategies were estimated to be USD 7322.33/QALY and USD 6289.59/QALY, both showing cost-effectiveness (Table S3). In contrast, when the vaccine price was USD 124.20 and USD 138.00, the ICER for the vaccination without screening and vaccination following screening strategies surpassed the WTP threshold, respectively, suggesting cost-ineffectiveness.

#### 3.4.3. Vaccine Dosing Schedules

Compared to fully receiving 2-dose and partially receiving 3-dose hepatitis E vaccine schedules, fully receiving 3-dose schedule prevented more HEV-related cases (Table S4). However, fully receiving 2-dose and partially receiving 3-dose schedules were cost-effective, regardless of the vaccination without screening or following screening; for the fully receiving 3-dose schedule, it was only cost-effective with the vaccination following the screening strategy (Table S5).

## 4. Discussion

Swine is a significant host for HEV and can act as a source of zoonotic transmission to humans [47]. Swine workers face a higher risk of HEV infection due to the nature of the work environment, which remains a significant public health concern [48]. Therefore, our study comprehensively evaluated the health and economic impact of hepatitis E vaccination strategies in swine workers in China. It showed that vaccination without screening and vaccination following screening strategies were both cost-effective and significantly reduced HEV-related cases compared to no vaccination. Moreover, despite the slightly higher incremental cost, hepatitis E vaccination following screening more effectively reduced HEV-related cases and minimized productivity loss, suggesting an optimal strategy. Previous studies also found that combining vaccination and screening was potentially cost-effective in multiple populations at risk of serious HEV infection, such as the elderly, hepatitis B patients, and women of childbearing age [15–17]. A recent modeling study further emphasized the effectiveness of interrupting the swine-to-person transmission route, vaccination, and shortening the infectious period in controlling HEV spread [49]. Therefore, hepatitis E vaccination is crucial to HEV prevention, routine water, sanitation and hygiene measures.

We conducted the sensitivity analysis to determine the possible factors affecting the ICER, such as the discount rate, utility of asymptomatic cases, and probability of symptomatic cases. The discount rate converts future costs and benefits into present values. A low discount rate increases the present value of future health benefits and makes current costs relatively lower, which reduces the ICER [50]. The utility of asymptomatic cases refers to the quality of life in individuals with asymptomatic HEV infection. The higher utility indicated that the impact of HEV infection on quality of life was minimal, which in turn reduced the expected cost-effectiveness of hepatitis E vaccination. Similarly, as the probability of symptomatic cases increased, more individuals with HEV infection needed treatment, which improved health outcomes and reduced the ICER [51]. This indicated vaccination strategies were more cost-effective compared to no vaccination, as early vaccination reduced treatment costs and improved overall cost-effectiveness. However, previous studies documented that vaccine price, vaccine efficacy, and decay of vaccine-induced immunity had the most significant impact on ICER [16]. Further research is crucial to better understand how factors such as occupational exposure, immune status, and other variables influence vaccine-induced immunity in specific populations. Considering the variations in the model design and parameter inputs, it remained significant to conclude a reasonable interpretation of the findings in the cost-effectiveness analysis in different contexts.

Moreover, by using the cost-effectiveness acceptability curve, we found that no vaccination strategy was more likely to be cost-effective at a low WTP threshold. As the WTP threshold increased, the probability of hepatitis E vaccination following the screening strategy being cost-effective increased. This was consistent with the results of the cost-effectiveness scatterplots. Compared to no vaccination, the proportion of scatter points on either side of the GDP threshold line was roughly equal. However, Cui et al. [16] conducted a PSA on chronic hepatitis B patients and found that, compared to no vaccination, 79.7% and 99.9% of the scatter points for the two vaccination strategies were below the GDP threshold line, indicating that vaccination would have been cost-effective for this population. We further found that the vaccination following screening strategy was superior to the vaccination without screening strategy. This finding was consistent with our base case analysis.

We also conducted the scenario analysis to determine the potential impact on the cost-effectiveness of hepatitis E vaccination in swine workers. Vaccination age, vaccine price, and dosing schedule were modifiable factors in health economic evaluations. First, hepatitis E vaccination starting at an earlier age proved to be more effective in preventing HEV-related cases and offered better cost-effectiveness compared to vaccination at a later age. Early vaccination not only reduces healthcare costs by preventing future infections and complications but also increases productivity by reducing illness-related workdays, resulting in significant long-term economic advantages. Policymakers can encourage swine workers of different ages to get vaccinated against HEV as soon as possible, taking into account regional epidemic status. Second, the high price of the hepatitis E vaccine in the Chinese market, which might be attributable to development costs and limited market, reduced the vaccination coverage [52]. However, as the coverage increases, the price might be expected to decrease, further promoting vaccine uptake. This price reduction could be achieved through government subsidies or large-scale production, both of which would lower costs and make the vaccine more accessible. In our scenario analysis, lowering the price of the hepatitis E vaccine reduced the ICER and improved the cost-effectiveness. Third, current studies showed that a 3-dose schedule (0, 1, and 6 months) provided optimal protection for at least 10 years [13, 53]. However, in resource-limited settings, the 3-dose schedule is costly and difficult to implement, particularly during outbreaks. A pilot trial of 2-dose schedule in Bangladesh confirmed that almost all participants maintained anti-HEV antibodies 2 years postvaccination [30]. The hepatitis E vaccination campaign in South Sudan in 2022 also demonstrated good tolerability of the vaccine [54]. Recently, the WHO reported 2092 suspected cases and seven deaths in a hepatitis E outbreak in eastern Chad, primarily in refugee camps [45]. Therefore, the SAGE recommended the 2-dose schedule (0, 1 month) for individuals in FCV settings instead of the 3-dose schedule [46]. Our study indicated that fully receiving 2-dose schedule was the most cost-effective strategy for swine workers. It would provide more evidence and insights into the cost-effectiveness of the 2-dose schedule. However, there were still several practical challenges. Individual immune responses may vary, potentially resulting in shorter immunity compared to the 3-dose schedule. The shorter interval between doses may also raise public concerns about long-term effectiveness and affect vaccination adherence. Additionally, during large-scale vaccination efforts, especially in outbreak situations, resource allocation and ensuring a stable vaccine supply were crucial for success.

This study has some limitations. First, most parameters in the model were derived from current epidemiological and disease burden studies in China. However, the availability of such studies was limited, and the reported data varied considerably across sources, which may affect the robustness of ICER estimates. Second, our model relied on simplified assumptions and did not consider postvaccination population movement or possible herd immunity effects. Markov model is inherently a static framework; it does not capture the dynamic transmission of hepatitis E or the potential impact of regional population shifts. Third, the efficacy data for the hepatitis E vaccine Hecolin are primarily derived from clinical trials conducted in China, where HEV genotype 4 is the most prevalent strain. While the vaccine has demonstrated cross-protection against other genotypes, further studies are needed to confirm its effectiveness across diverse HEV strains. However, this study still has strengths. We optimized the model design, parameter acquisition, and scenario analyses based on previously published studies. Moreover, we included intent to receive the hepatitis E vaccine and a number of vaccine doses administered to simulate vaccination coverage with different strategies. We also explored the progression of hepatitis E after infection and estimated the number of various HEV-related cases, including outpatient and inpatient cases, ALF, and deaths, to comprehensively assess the effects of vaccination.

## 5. Conclusions

This study demonstrated that hepatitis E vaccination following screening was the most cost-effective strategy for preventing hepatitis E among swine workers. Vaccination starting at an earlier age and reducing the vaccine price may achieve the cost-effectiveness and then optimal health protection, especially in resource-limited settings. Additionally, during hepatitis E outbreaks, 2-dose schedule may be prioritized to achieve rapid health protection.

## Figures and Tables

**Table 1:** Base-case values, ranges, and distributions of parameters in the decision tree-Markov model.

Parameter	Base-case value	Range	Distribution	Reference
Probability				
Infection	0.0367	±20%	Triangular	[21]
Symptomatic infection	0.165	0.05–0.33	Triangular	[1, 2, 22]
Hospitalization	0.328	0.273–0.391	Triangular	[12, 14]
Develop ALF	0.100	0.001–0.280	Beta	[23]
Die of ALF	0.230	0.140–0.340	Beta	[23, 24]
All-cause mortality (‰)	—	—	—	National Bureau of Statistics of China [25]
18–19 years	0.29	—	—	—
20–24 years	0.33	—	—	—
25–29 years	0.39	—	—	—
30–34 years	0.51	—	—	—
35–39 years	0.78	—	—	—
40–44 years	1.25	—	—	—
45–49 years	1.94	—	—	—
50–54 years	2.99	—	—	—
55–59 years	4.51	—	—	—
60 years	7.48	—	—	—
Natural immunity rate	—	—	—	[10, 26, 27]
16–20 years	0.214	± 20%	Triangular	—
21–30 years	0.284	± 20%	Triangular	—
31–40 years	0.513	± 20%	Triangular	—
41–50 years	0.417	± 20%	Triangular	—
51–60 years	0.478	± 20%	Triangular	—
Natural immunity decay	0.0483	± 20%	Triangular	[28]
Vaccination coverage				
3-dose	0.865	0.7785–0.9515	Triangular	[13, 29]
2-dose	0.0669	0.0648–0.0690	Triangular	[13, 29]
1-dose	0.0688	0.0667–0.0709	Triangular	[13, 29]
Vaccine efficacy				
3-dose	0.866	0.730–0.941	Triangular	[13, 14]
2-dose	0.858	0.434–0.997	Triangular	[13, 30]
1-dose	0.834	0.367–0.996	Triangular	[12]
Vaccine immunity decay (%/year)	0.0159	0.0052–0.0265	Triangular	[13]
Willing to be vaccinated after screening	0.80	0.75–0.95	Triangular	[29, 31]
Willing to be vaccinated without screening	0.54	0.492–0.588	Triangular	[32]
Cost (USD)				
Vaccine price (/dose)	106.26	—	—	Shanghai Xuhui District Government [33]
Screening cost (/time)	4.14	—	—	—
Vaccination and management cost of vaccination clinics (/dose)	3.86	—	—	Zhejiang Provincial Price Bureau [34]
Indirect cost of vaccination	—	—	—	National Bureau of Statistics of China [35]
3-dose	213.57	106.79–320.36	Triangular	—
2-dose	142.38	71.19–213.57	Triangular	—
1-dose	71.19	35.60–106.79	Triangular	—
Outpatient cases	116.00	39.40–487.80	Gamma	[36]
Inpatients cases	3272.10	1689.20–4932.50	Gamma	[36]
ALF cases	4514.44	2749.68–6298.22	Gamma	[37]
Deaths	10,149.45	3452.39–16846.51	Gamma	[38]
Discount rate (%)	5	0–10	Triangular	Default
GDP per capita	12,325.24	—	—	National Bureau of Statistics of China [39]
Utilities (QALY)				
Health	1	—	—	Default
Asymptomatic infection	0.95	0.90–1.00	Beta	[40, 41]
Outpatient cases	0.80	0.70–1.0	Beta	[36]
Inpatients cases	0.72	0.54–0.90	Beta	[38]
ALF cases	0.38	0.36–0.41	Beta	[42]
Deaths	0	—	—	Default
Retiring age	60	—	—	The State Council of the People's Republic of China [43]

Abbreviations: ALF, acute liver failure; QALY, quality-adjusted life year.

**Table 2:** Hepatitis E vaccination doses and associated costs with different vaccination strategies in swine workers.

Number of doses	Vaccination without screening		Vaccination following screening		Difference (USD)
	Total doses administered	Total vaccine-related costs (USD)	Total doses administered	Total vaccine-related costs (USD)	
3	140,017	16,794,057.73	167,398	20,309,237.74	—
2	7225	866,626.61	8638	1,053,981.19	—
1	3715	445,601.09	4442	551,129.61	—
0	0	0	0	146,721.60	—
Total	150,957	18,106,285.43	180,477	22,061,070.13	3,954,784.71

**Table 3:** Cost-effectiveness analysis of hepatitis E vaccination strategies and no vaccination in swine workers.

Outcome variables	Vaccination without screening	Vaccination following screening	No vaccination
Number of outpatient cases	6802	6111	9932
Number of outpatient cases avoided	3129	3821	—
Number of inpatient cases	2645	2376	3862
Number of inpatient cases avoided	1217	1486	—
Number of acute liver failures	244	226	367
Number of acute liver failures avoided	123	141	—
Number of deaths	52	46	75
Number of deaths avoided	24	29	—
Cost	22,370,398.60	25,383,253.82	6,714,738.06
Incremental cost	15,655,660.54	18,668,515.76	—
QALY	1,792,591.57	1,793,120.65	1,791,221.65
Incremental QALY	1369.92	1899.00	—
ICER^a^	11,428.16	9830.71	—
ICER^b^	5694.52	—	—

Abbreviations: ICER, incremental cost-effectiveness ratio; QALY, quality-adjusted life year.

^a^Hepatitis E vaccination strategies (vaccination without screening and vaccination following screening) versus no vaccination.

^b^Hepatitis E vaccination following screening strategy versus vaccination without screening strategy.

## Data Availability

The datasets used and/or analyzed during the current study are available from the corresponding author upon reasonable request.

## Nomenclature

HEV: Hepatitis E virus
WHO: World Health Organization
ALF: Acute liver failure
WTP: Willingness-to-pay
QALY: Quality-adjusted life year
ICER: Incremental cost-effectiveness ratio
PSAs: Probabilistic sensitivity analyses
SAGE: Strategic Advisory Group of Experts
FCV: Fragile, conflict-affected, and vulnerable.
