# Supplementary material for: Cost-Effectiveness Analysis of Hepatitis E Vaccination Strategies for Swine Workers
Source: Transbound Emerg Dis. 2025 Mar 10;2025:9371055. doi: 10.1155/tbed/9371055 (PMC12017058; doi:10.1155/tbed/9371055)
Supplement: Supporting Information — Additional supporting tables and figures can be found in the Supporting Information. [file 9371055.f1.docx]

**Supplementary Materials**

**TABLE S1** Number of HEV-related cases avoided by hepatitis E vaccination starting at various ages

| Vaccination starting age (years) | Vaccination without screening vs. no vaccination | | | | Vaccination following screening vs. no vaccination | | | |
| --- | --- | --- | --- | --- | --- | --- | --- | --- |
|  | Number of outpatient cases avoided | Number of inpatient cases avoided | Number of acute liver failures avoided | Number of deaths avoided | Number of outpatient cases avoided | Number of inpatient cases avoided | Number of acute liver failures avoided | Number of deaths avoided |
| 16-60 | 3129 | 1217 | 123 | 24 | 3821 | 1486 | 141 | 29 |
| 20-60 | 2882 | 1121 | 106 | 22 | 3513 | 1366 | 130 | 27 |
| 30-60 | 2208 | 859 | 82 | 17 | 2724 | 1060 | 101 | 21 |
| 40-60 | 1662 | 646 | 61 | 13 | 2163 | 841 | 80 | 16 |
| 50-60 | 877 | 341 | 32 | 7 | 1210 | 471 | 45 | 9 |

HEV, hepatitis E virus.

**TABLE S2** Cost-effectiveness analysis of hepatitis E vaccination starting at various ages

| Vaccination starting age (years) | Vaccination without screening vs. no vaccination | | | | | | Vaccination following screening vs. no vaccination | | | | | |
| --- | --- | --- | --- | --- | --- | --- | --- | --- | --- | --- | --- | --- |
|  | Incremental cost (USD) | ICER (USD/outpatient case avoided) | ICER (USD/inpatient case avoided) | ICER (USD/acute liver failure case avoided) | ICER (USD/death avoided) | ICER (USD/QALY) | Incremental cost (USD) | ICER (USD/outpatient case avoided) | ICER (USD/inpatient case avoided) | ICER (USD/acute liver failure case avoided) | ICER (USD/death avoided) | ICER (USD/QALY) |
| 16-60 | 15655660.54 | 5002.99 | 12864.78 | 127686.65 | 660297.79 | 11563.74 | 18668515.76 | 4886.01 | 12564.11 | 132250.75 | 645076.56 | 9939.16 |
| 20-60 | 15692943.13 | 5445.63 | 14003.18 | 147420.79 | 718870.51 | 11718.84 | 18714286.40 | 5326.67 | 13697.16 | 144177.86 | 703016.02 | 11063.21 |
| 30-60 | 15951181.96 | 7225.22 | 18579.43 | 195576.04 | 954588.99 | 13662.57 | 17666512.85 | 6484.36 | 16674.07 | 175524.22 | 856350.60 | 11048.46 |
| 40-60 | 16536493.47 | 9950.17 | 25585.22 | 269324.00 | 1314506.64 | 20098.81 | 14406375.67 | 6661.01 | 17128.22 | 180305.08 | 878973.50 | 13381.98 |
| 50-60 | 17004276.36 | 19397.55 | 49877.61 | 525147.51 | 2564747.57 | 30644.96 | 16546086.35 | 13672.19 | 35156.57 | 370075.74 | 1804371.47 | 21251.89 |

ICER, Incremental cost-effectiveness ratio. QALY, quality-adjusted life year.

**TABLE S3** Cost-effectiveness analysis of hepatitis E vaccination at various vaccine prices

| Vaccine price (USD)^1^ | Vaccination without screening vs. no vaccination | | | | | | Vaccination following screening vs. no vaccination | | | | | |
| --- | --- | --- | --- | --- | --- | --- | --- | --- | --- | --- | --- | --- |
|  | Incremental cost (USD) | ICER (USD/outpatient case avoided) | ICER (USD/inpatient case avoided) | ICER (USD/acute liver failure case avoided) | ICER (USD/death avoided) | ICER (USD/QALY) | Incremental cost (USD) | ICER (USD/outpatient case avoided) | ICER (USD/inpatient case avoided) | ICER (USD/acute liver failure case avoided) | ICER (USD/death avoided) | ICER (USD/QALY) |
| 69.00 | 10031002.72 | 3205.55 | 8242.81 | 81812.27 | 423070.55 | 7322.33 | 11943924.86 | 3126.02 | 8038.39 | 84612.67 | 412713.37 | 6289.59 |
| 82.80 | 12114209.32 | 3871.27 | 9954.65 | 98802.78 | 510932.49 | 8843.00 | 14434514.08 | 3777.87 | 9714.59 | 102256.40 | 498773.81 | 7601.11 |
| 96.60 | 14197415.92 | 4536.99 | 11666.49 | 115793.30 | 598794.43 | 10363.68 | 16925103.30 | 4429.72 | 11390.78 | 119900.14 | 584834.25 | 8912.64 |
| 106.26 | 15655660.54 | 5002.99 | 12864.78 | 127686.65 | 660297.79 | 11428.16 | 18668515.76 | 4886.01 | 12564.11 | 132250.75 | 645076.56 | 9830.71 |
| 110.40 | 16280622.52 | 5202.71 | 13378.33 | 132783.81 | 686656.37 | 11884.36 | 19415692.53 | 5081.56 | 13066.97 | 137543.87 | 670894.70 | 10224.17 |
| 124.20 | 18363829.12 | 5868.43 | 15090.17 | 149774.32 | 774518.31 | 13405.04 | 21906281.75 | 5733.41 | 14743.17 | 155187.60 | 756955.14 | 11535.69 |
| 138.00 | 20447035.72 | 6534.14 | 16802.01 | 166764.83 | 862380.25 | 14925.72 | 24396871.00 | 6385.26 | 16419.36 | 172831.33 | 843015.58 | 12847.22 |
| 151.80 | 22530242.32 | 7199.86 | 18513.85 | 183755.34 | 950242.19 | 16446.39 | 26887460.20 | 7037.11 | 18095.55 | 190475.07 | 929076.03 | 14158.75 |

^1^ The hepatitis E vaccine prices of USD 69.00, 82.80, 96.60, 106.26, 110.40, 124.20, 138.00, and 151.80 corresponded to CNY 500, 600, 700, 770, 800, 900, 1000, and 1100, respectively.

ICER, Incremental cost-effectiveness ratio.

**TABLE S4** Number of HEV-related cases avoided by hepatitis E vaccine dosing schedules

| Vaccine dosing schedules | Vaccination without screening vs. no vaccination | | | | Vaccination following screening vs. no vaccination | | | |
| --- | --- | --- | --- | --- | --- | --- | --- | --- |
|  | Number of outpatient cases avoided | Number of inpatient cases avoided | Number of acute liver failures avoided | Number of deaths avoided | Number of outpatient cases avoided | Number of inpatient cases avoided | Number of acute liver failures avoided | Number of deaths avoided |
| Fully receiving 3-dose | 3139 | 1221 | 116 | 24 | 3833 | 1491 | 142 | 29 |
| Partially receiving 3-dose | 3129 | 1217 | 123 | 24 | 3821 | 1486 | 141 | 29 |
| Fully receiving 2-dose | 3110 | 1210 | 115 | 24 | 3798 | 1477 | 140 | 29 |

HEV, hepatitis E virus.

**TABLE S5** Cost-effectiveness analysis of hepatitis E vaccine dosing schedules

| Vaccination dose strategy | Vaccination without screening vs. no vaccination | | | | | | Vaccination following screening vs. no vaccination | | | | | |
| --- | --- | --- | --- | --- | --- | --- | --- | --- | --- | --- | --- | --- |
|  | Incremental cost (USD) | ICER (USD/outpatient case avoided) | ICER (USD/inpatient case avoided) | ICER (USD/acute liver failure case avoided) | ICER (USD/death avoided) | ICER (USD/QALY) | Incremental cost (USD) | ICER (USD/outpatient case avoided) | ICER (USD/inpatient case avoided) | ICER (USD/acute liver failure case avoided) | ICER (USD/death avoided) | ICER (USD/QALY) |
| Fully receiving 3-dose | 16972425.9 | 5406.66 | 13902.82 | 146351.87 | 714027.17 | 12350.23 | 20241315.02 | 5280.90 | 13579.49 | 142937.05 | 697255.08 | 10625.25 |
| Partially receiving 3-dose | 15655660.54 | 5002.99 | 12864.78 | 127686.65 | 660297.79 | 11428.16 | 18668515.76 | 4886.01 | 12564.11 | 132250.75 | 645076.56 | 9830.71 |
| Fully receiving 2-dose | 10518376.24 | 3381.94 | 8696.32 | 91543.74 | 446450.60 | 7725.18 | 12262149.58 | 3229.01 | 8303.19 | 87399.50 | 426361.25 | 6496.78 |

ICER, Incremental Cost-Effectiveness Ratio


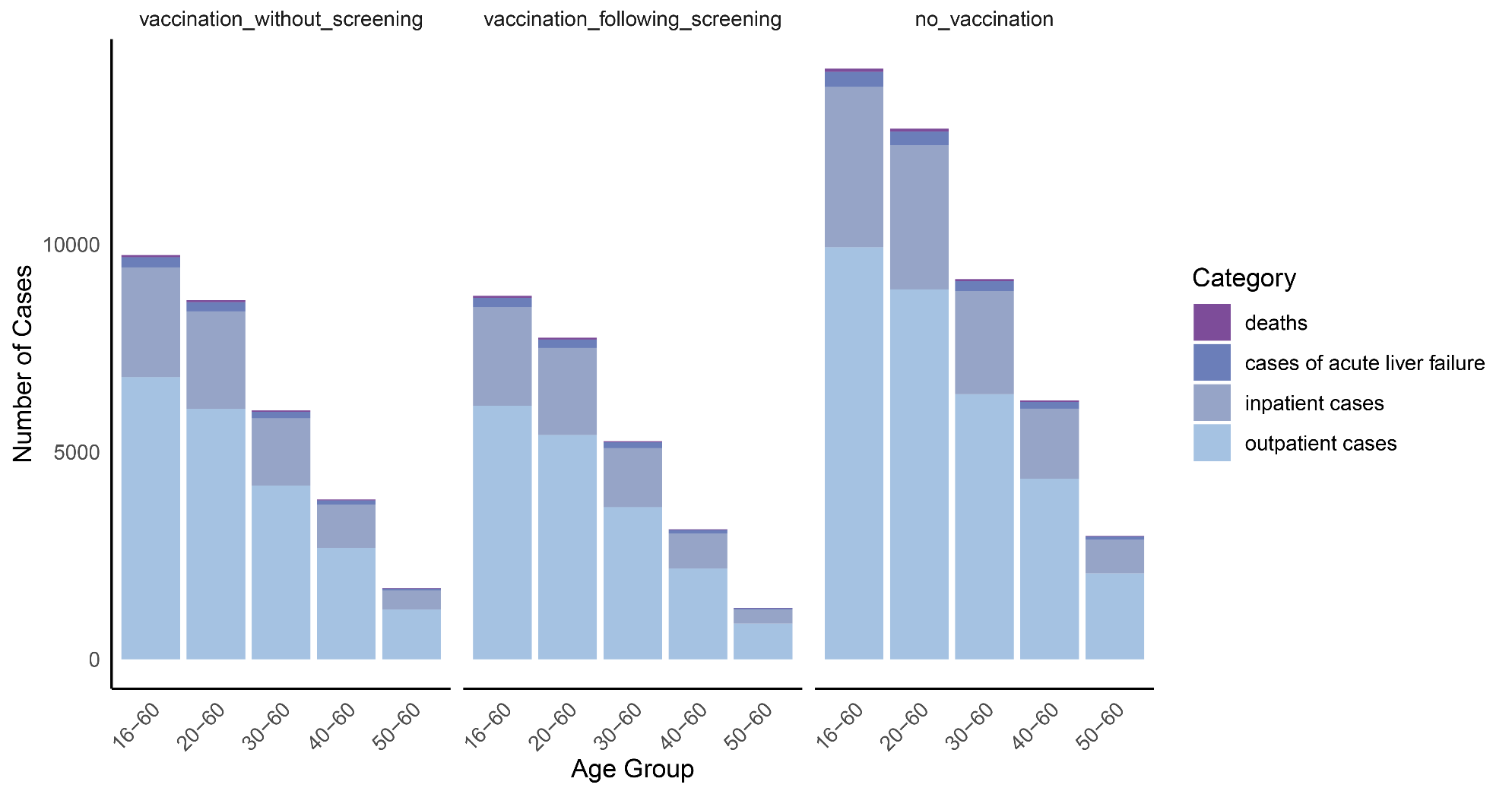


**FIGURE S1** Number of HEV-related cases by two hepatitis E vaccination strategies and no vaccination starting at various ages. HEV, hepatitis E virus.
